# An easy and safe training method for trunk function improves mobility in total knee arthroplasty patients: A quasi-randomized controlled trial

**DOI:** 10.1371/journal.pone.0204884

**Published:** 2018-10-04

**Authors:** Yuki Sano, Akira Iwata, Hideyuki Wanaka, Mina Matsui, Saki Yamamoto, Junichiro Koyanagi, Hiroshi Iwata

**Affiliations:** 1 Department of Physical Therapy, Faculty of Comprehensive Rehabilitation, Osaka Prefecture University, Habikino, Osaka, Japan; 2 Department of Rehabilitation, Osaka General Medical Center, Osaka, Osaka, Japan; 3 Department of Orthopaedic Surgery, Osaka General Medical Center, Osaka, Osaka, Japan; 4 Department of Cardiovascular Medicine, Juntendo University Graduate School of Medicine, Bunkyo-ku, Tokyo, Japan; Universitat de Valencia, SPAIN

## Abstract

**Objective:**

Total knee arthroplasty (TKA) is aimed mainly at reducing pain and restoring mobility. However, mobility deficits can persist even longer than 1 year. The trunk function and movement velocity of any region have been recently recognized to be critical for determining mobility in older people. Therefore, the main goal of this quasi-randomized trial is to clarify the effectiveness of a novel training method, the seated side tapping (SST) training, for improving mobility by focusing on movement velocity of trunk function in the short term after TKA.

**Methods:**

SST training consists of side trunk movements repeated as quickly as possible in a seated position. All participants after TKA were randomly assigned to the SST training group (n = 37) or control training group (n = 38). The participants in the SST group performed SST training plus the standard rehabilitation program 5 days per week for 3 weeks after TKA, while the control group performed only the standard rehabilitation programs. The primary outcome was the effect of SST training on mobility, indicated by gait speed and the timed up and go test (TUG) time. Measurements were performed before and 1, 2, and 3 weeks after surgery.

**Results:**

At all-time points, the patients in the SST group showed significantly better mobility, despite that knee function, represented by muscle strength, range of motion, and degree of pain at the knee joint, was similar in both groups. The difference in gait speed between the groups was >0.1 m/s at all time points, which is clinically significant.

**Conclusion:**

SST training significantly improved patients’ mobility within 3 weeks after TKA, despite that no additional benefit was observed in knee function. The findings in this study indicate that SST training may be considered as a part of the rehabilitation program after TKA, although further evaluation of its long-term effectiveness is needed.

**Trial registration:**

University Hospital Medical Information Network Clinical Trials Registry (UMIN-CTR; UMIN000027909).

## Introduction

Knee osteoarthritis (OA) is one of the most frequent causes of disability in older people, and total knee arthroplasty (TKA) is usually indicated as a surgical intervention for end-stage knee OA with the aims of pain relief, deformity correction, improvement of knee function, and restoration of locomotor function [1–3]. Even though TKA reduces pain and improves overall health-related quality of life in 90% of patients with a high patient satisfaction rate [4], recovery of locomotor function after TKA to a normal level is not common [5, 6]. A previous study demonstrated that patients 1 month after TKA had a 50% longer timed up and go test (TUG) time and walked 40% shorter distance in the 6-minute walking test than those before surgery [5]. Another study also showed that the gait speed of patients who underwent TKA was not fully recovered even at 1 year after TKA as compared with the age-matched controls [6]. To maintain or restore mobility after TKA, rehabilitation programs thus far have mainly focused on knee function, represented by knee joint pain [7], muscle strength of the quadriceps and hamstrings [8], and ranges of motion of knee flexion and extension [9].

Trunk function and the movement velocity of any body parts have recently been recognized as important factors for determining gait performance in older people. Trunk function has been shown to be of importance for balance control during walking [10], and core stability training consisting of trunk exercises was reported to be effective at improving gait speed in community-dwelling older adults [11]. In addition, in patients who had undergone TKA, trunk function compensates for knee function during walking [12].

The movement velocity of the lower limbs has also been shown to be a more reliable predictor of performance of lower-intensity functional tasks such as gait speed than muscle strength in community-dwelling older people [13]. High-speed resistance training is more efficient at increasing gait speed in healthy older people than low-speed resistance training [14]. Muscle power, the product of strength and movement velocity, is more closely associated with gait speed early after TKA than with strength alone [15].

We previously showed that the seated side tapping (SST) test, which measures the time in seconds needed to move the body laterally from one side to the other 10 times as quickly as possible in a seated position, was closely associated with mobility in frail and healthy older people [16, 17]. On the basis of such findings that the SST test reflects the ability of how quick a participant can move his or her trunk, we hypothesized that the SST test would be suitable for a rehabilitation program for improvement in gait performance after TKA. Therefore, the purpose of this study was to assess the effects of SST as a training program (“SST training”) on the prognosis of mobility in patients who had undergone TKA.

## Materials and methods

### Participants

The present study was designed as a prospective, non-blinded, quasi-randomized controlled trial. Patients (n = 105) were recruited between May 1, 2012, and August 1, 2013, and followed up until August 31, 2013. All subjects scheduled to undergo TKA for knee OA at Osaka General Medical Center were evaluated for their eligibility to be included in this study in accordance with inclusion/exclusion criteria. The inclusion criteria were as follows: 1) aged ≥60 years and 2) had the ability to walk ≥10 m without assistance 1 week after TKA. Participants were excluded if they had any medical or neurological problem that would affect their ability to complete this trial, such as stroke, cardiac insufficiency, or acute respiratory failure.

**Fig 1:**
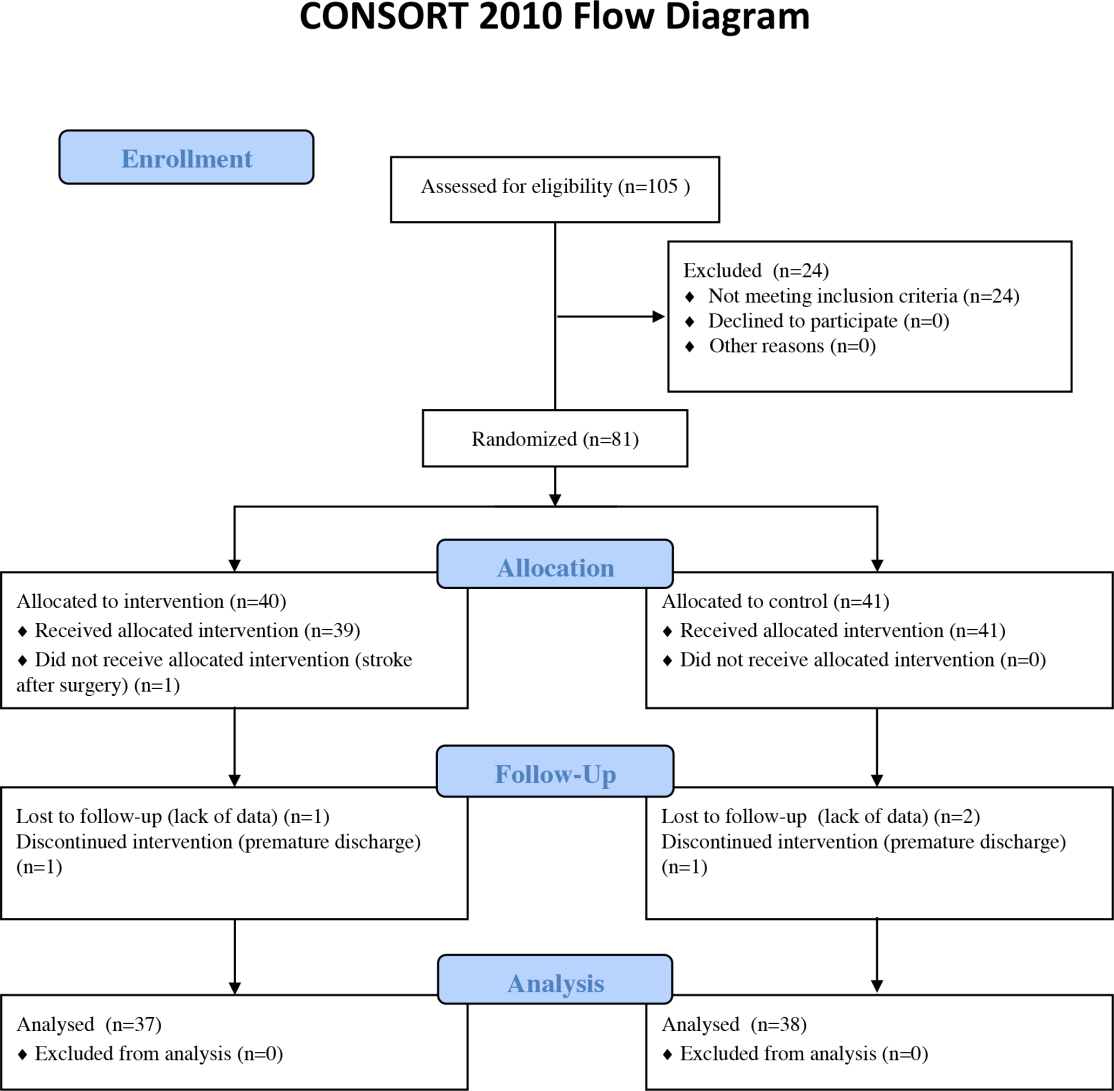
CONSORT flow diagram showing the enrollment and progress of the study participants.

This study was approved by the ethics committees of Osaka Prefecture University (approval No. 2012-PT04) and Osaka General Medical Center (approval No. 23–0917). Written informed consent was obtained from all the participants. The protocols for this study and supporting CONSORT checklist are available as supporting information (Fig 1, S1 Fig, S1 Table, and S1 File).

Three experienced surgeons with >10 years of experience and a high degree of expertise in joint replacement surgery performed all the surgeries. The operations were performed with the use of an air tourniquet, which was inflated during draping and released after dressing. A standard medial parapatellar approach was used, and both the anterior and posterior cruciate ligaments were resected.

This study was registered in the University Hospital Medical Information Network Clinical Trials Registry (UMIN-CTR; UMIN000027909). The authors confirmed that all ongoing and related trials for this intervention are registered. The major reason for not registering this study before the enrolment of participants was that we were not aware of the need to register it at the start of the study.

### Randomization

A quasi-randomized design was used to evaluate the effectiveness of SST training. The participants who fulfilled the inclusion criteria were divided alternately into the SST and control group according to the order of operation.

### Rehabilitation and training after TKA

A standard rehabilitation program performed 5 days a week by a physical therapist was initiated the day after TKA and continued for 3 weeks. Full weight bearing was allowed from the first postoperative day. At 2 days after surgery, all the participants started standing and performing gait exercises using parallel bars or walking aids. At the same time, manual therapy techniques, which consisted of muscle stretching and soft tissue mobilization, were applied primarily to the knee and surrounding structures. In addition to receiving manual therapy, all the participants received active and passive range of motion (ROM) exercises, muscle strength training, up and down stairs exercise, and riding a stationary bicycle.

### Intervention

**Fig 2:**
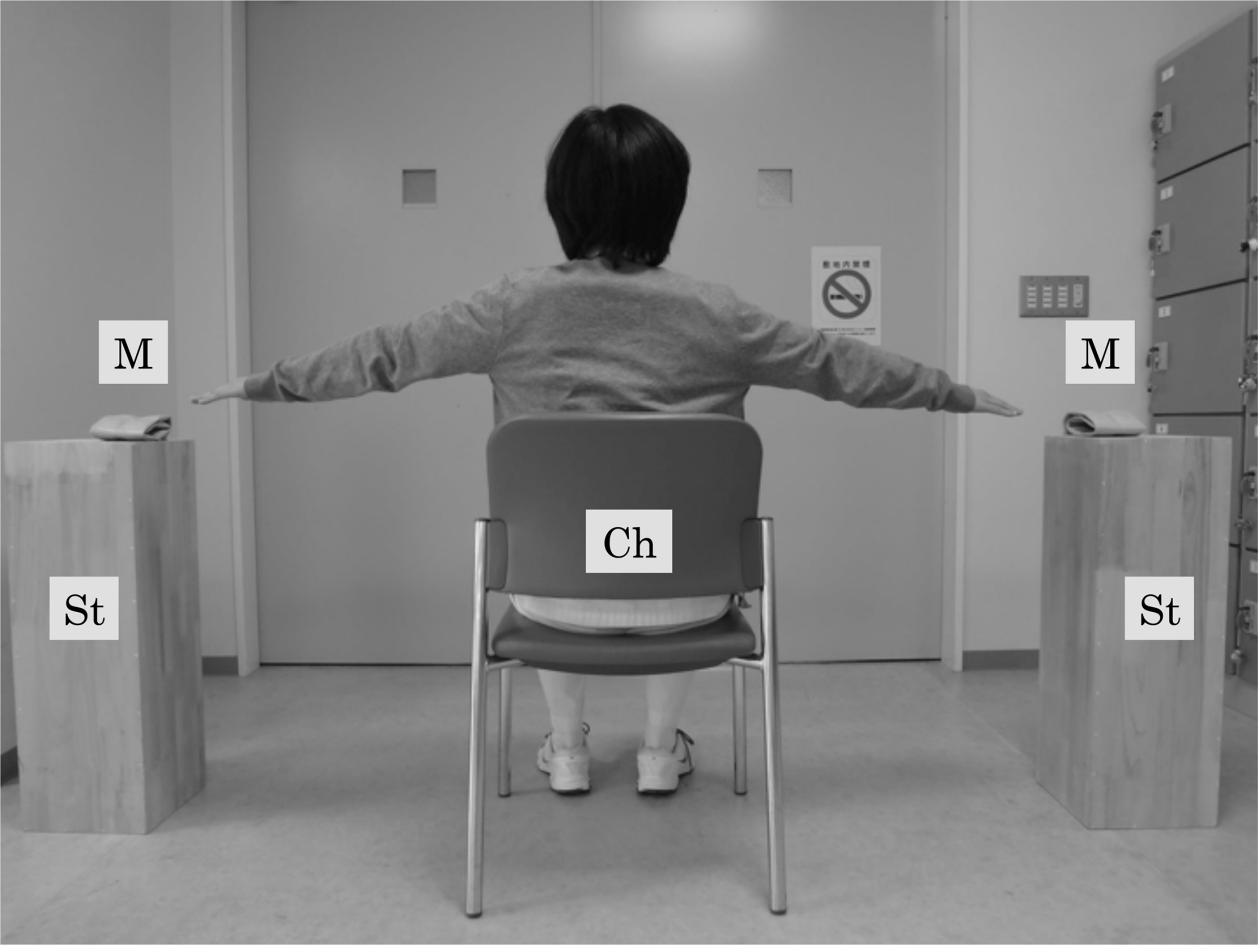
Measurement apparatus used in seated side tapping training. M: marker (diameter, 10 cm), St: stand (height, 72 cm), Ch: chair (height, 41 cm from a floor).

The patients in the SST group performed SST training in addition to the standard inpatient rehabilitation program as described earlier. The apparatus required for SST training was described in detail elsewhere [16]. Briefly, we placed a stand on either side of the chair that the participant was sitting on, and a marker was placed on each stand (Fig 2). The participants raised their arms to shoulder height, and the therapist moved the stands to positions 10 cm from their fingertips. The participants were instructed to tap the markers alternately 10 times as quickly as possible. No restrictions on foot position were imposed. One set of SST training included 5 repetitions with approximately 10-second intervals, and the total time of one set of training, including preparation, was 3 minutes per day.

### Measures

The primary outcome was to evaluate the effect of SST training on mobility, and the secondary outcome was knee function. These clinical data were prospectively collected for all the participants preoperatively and postoperatively at 1, 2, and 3 weeks.

#### Mobility

Gait speed and TUG time were measured as indicators of mobility. A walking aid was provided when the physical therapist concluded it was needed for the safety of the patient. Gait speed was determined by instructing the participants to walk at their usual pace over an 8-m walkway. The initial and final 1.5-m sections were not timed to allow for acceleration and deceleration, so only the time needed to walk the middle 5 m of the walkway was measured.

TUG was performed as previously reported by Podsiadlo and Richardson [18]. The physical therapist instructed the participants to stand up, walk 3 m at their usual speed, cross a line, turn around, walk back, and sit down on the same chair. In both mobility tests, measurements were performed twice by using a stopwatch, and the faster of the two was used for the subsequent analysis.

#### Knee function

Passive knee flexion and extension ROM were measured with the participants in a supine lying position, by using a standard long-arm goniometer. Quadriceps and hamstring muscle strengths were measured with the participants seated with their knee flexed to 90° on a handheld dynamometer [19]. These tests were performed twice, and the higher value was recorded. Pain was assessed just after the gait speed test by using a visual analogue scale [20].

### Sample size

The sample size was calculated using G*Power v3.1. We assumed that the SST group could achieve an increase in gait speed of at least 0.1 m/sec as compared with the control group because a 0.1-m/s difference in gait speed is considered a substantial meaningful change in older adults [21, 22]. An a priori power calculation was performed for the Student *t* test. To achieve 80% power to detect meaningful differences at the 5% level (two-tailed), 37 patients in each group would be needed. Allowing for a 20% dropout rate, 95 patients were required for enrollment in this study.

### Statistical analysis

All continuous variables are expressed as means and standard deviations (SDs). A chi-square test was conducted to compare the proportion of participants in each group by each surgeon. A two-way repeated-measures analysis of variance was used to examine the interaction between the groups (SST and control groups) and time. Moreover, differences between the groups at each time point were analyzed using the Student *t* test, and p values were adjusted for multiple comparisons using the Benjamini-Hochberg false discovery rate correction [23]. If results of Mauchly’s test indicated violation of the sphericity assumption, degrees of freedom were corrected using the Greenhouse-Geisser estimates of sphericity. Effect size was calculated using Cohen’s *d* and interpreted as small (≥0.20), medium (≥0.50), or large (≥0.80) [24]. All analyses were performed using the SPSS software (version 21.0), and the level of statistical significance was set at 0.05.

## Results

A total of 105 participants provided informed consent for enrollment in this study before TKA (Fig 1). Among the participants enrolled, 24 were excluded from analysis because they could not walk without a walker for ≥10 m at 1 week after surgery. One participant in the SST group was excluded because of stroke after TKA, which required medical intervention. Two patients (1 in each group) did not complete the study course, as they were prematurely discharged at 2 weeks after TKA, which means that they had no data for the third week. Three participants were excluded for lack of data (1 in the SST group and 2 in the control group). After excluding 30 of the 105 original participants, 75 were finally included in the analysis. In this study, no adverse event caused by any rehabilitation training was reported. Among the 75 participants, 37 were assigned to the SST group and 38 to the control group. The proportions of participants by each surgeon were similar in each group, and no significant difference was observed in the chi-square test (p = 0.605; Table 1).

**Table 1:** Number of participants in the SST and control groups by each surgeon.

Surgeon	SST group (n = 37)	Control group (n = 38)
A	13	18
B	6	7
C	18	13

The background characteristics of the participants, such as age, sex, height, body weight, and body mass index, were similar between the two groups (Table 2). The mean age of the SST group (81.1% female) was 75.0 ± 6.4 years and that of the control group (78.9% female) was 75.8 ± 5.8 years.

**Table 2:** Baseline characteristics of the study subjects.

	SST group (n = 37)		Control group (n = 38)	
	Value	Range	Value	Range
Age (years)	75.0 ± 6.4	62 to 89	75.8 ± 5.8	64 to 88
Female, n (%)	30 (81.1)		30 (78.9)	
Height (cm)	151.1 ± 8.3	132.5 to 171.0	152.6 ± 6.5	138.5 to 172.0
Weight (kg)	59.4 ± 11.5	42.0 to 93.0	62.0 ± 9.7	43.3 to 83.0
Body mass index (kg/m^2^)	25.9 ± 3.5	19.7 to 34.0	26.6 ± 3.2	20.0 to 32.4
FTA (°)	183.1 ± 7.2	170 to 200	181.8 ± 8.3	167 to 205
JOA score	54.5 ± 8.9	35 to 70	54.2 ± 6.6	45 to 80
KL	3.4 ± 0.7	1 to 4	3.3 ± 0.7	1 to 4
FIM	122.8 ± 2.1	118 to 126	122.9 ± 1.6	119 to 126

Mean ± standard deviation (SD). FTA: femorotibial angle. JOA score: Japanese Orthopaedic Association score. KL: Kellgren-Lawrence grading scale. FIM: Functional Independence Measure

**Fig 3:**
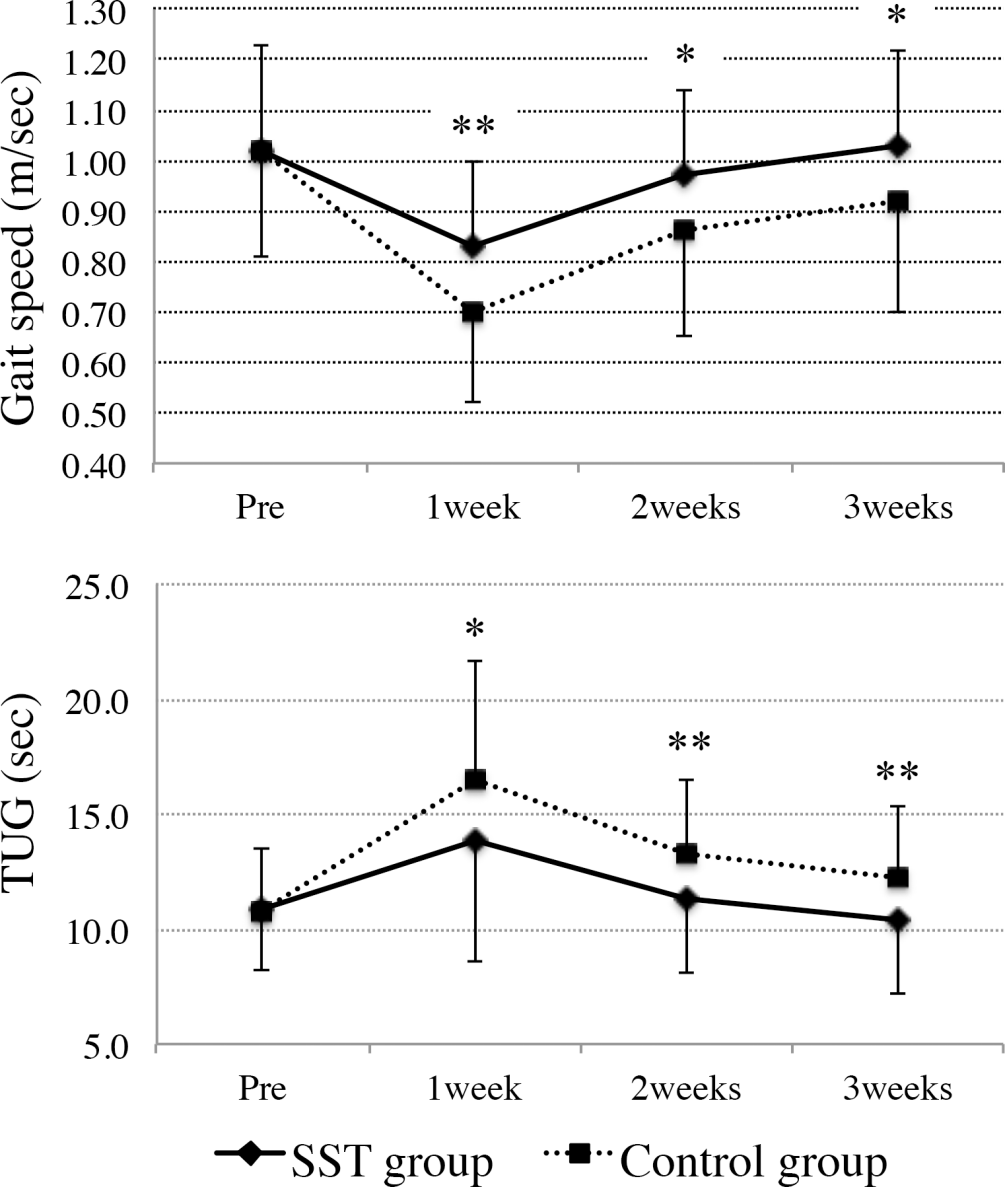
Comparison of gait speed and TUG time between the SST and control groups before surgery and at 1, 2, and 3 weeks after surgery. *p < 0.05, **p < 0.01 (adjusted for multiple comparisons using the Benjamini-Hochberg method).

Significant group × time interactions were observed for gait speed and the TUG time, but not for knee function (knee flexion and extension ROM, muscle strengths of the quadriceps and hamstrings, and knee pain; Table 3). Gait speed at all time points after TKA was significantly higher in the SST group than in the control group (Table 3, Fig 3). Similarly, the participants in the SST group had significantly shorter times for the TUG at all time points after surgery (Table 3, Fig 3). By contrast, the parameters representing knee function were not significantly different between the SST and control groups at all time points. Furthermore, the effect sizes of gait speed and TUG were medium at all time points after TKA (Table 3). Post hoc power analysis revealed powers of 0.89 (at 1 week), 0.72 (at 2 weeks), and 0.60 (at 3 weeks) for gait speed, and 0.78 (at 1 week), 0.87 (at 2 weeks), and 0.84 (at 3 weeks) for TUG time.

**Table 3:** Descriptive statistics and group comparisons of mobility and knee function.

	SST group (n = 37)			Control group (n = 38)			group × time interaction	t-test	t-test	Cohen's d
							*p*-Value	*p*-Value	*p*-Value*	
Gait speed							0.001			
Pre	1.02 ± 0.21			1.02 ± 0.21				0.936	0.936	-0.019
1 week	0.83 ± 0.17			0.70 ± 0.18				0.002	0.008	0.742
2 weeks	0.97 ± 0.17			0.86 ± 0.21				0.012	0.024	0.597
3 weeks	1.03 ± 0.19			0.92 ± 0.22				0.028	0.038	0.517
TUG							0.001			
Pre	10.94 ± 2.59			10.81 ± 2.71				0.828	0.828	0.050
1 week	13.78 ± 3.19			16.52 ± 5.19				0.008	0.010	-0.636
2 weeks	11.30 ± 2.31			13.33 ± 3.21				0.002	0.008	-0.725
3 weeks	10.44 ± 1.87			12.22 ± 3.15				0.004	0.008	-0.688
Flexion ROM (degrees)							0.364			
Pre	126.2 ± 12.2			125.4 ± 13.2				0.780	0.780	0.065
1 week	111.1 ± 9.2			110.1 ± 10.4				0.678	0.921	0.096
2 weeks	115.3 ± 8.7			116.1 ± 8.2				0.691	0.921	-0.092
3weeks	119.1 ± 8.2			121.1 ± 7.5				0.277	0.921	-0.253
Extension ROM (degrees)							0.823			
Pre	-8.1 ± 8.1			-8.7 ± 7.1				0.745	0.745	0.075
1 week	-6.4 ± 4.5			-7.6 ± 5.2				0.257	0.481	0.264
2 weeks	-4.7 ± 4.4			-5.8 ± 4.9				0.326	0.481	0.228
3 weeks	-3.8 ± 4.5			-4.7 ± 4.5				0.360	0.481	0.213
Quadriceps strength (kg)							0.883			
Pre	16.2 ± 7.1			16.0 ± 6.7				0.904	0.904	0.028
1 week	4.4 ± 2.0			4.5 ± 3.1				0.898	0.904	-0.030
2 weeks	6.4 ± 2.7			6.6 ± 2.8				0.812	0.904	-0.055
3 weeks	7.7 ± 2.9			7.8 ± 3.3				0.830	0.904	-0.050
Hamstrings strength (kg)							0.654			
Pre	9.5 ± 3.8			9.2 ± 4.5				0.780	0.811	0.065
1 week	6.1 ± 2.9			5.7 ± 3.0				0.528	0.811	0.147
2 weeks	6.2 ± 1.9			6.3 ± 2.8				0.811	0.811	-0.056
3 weeks	6.7 ± 2.4			6.9 ± 2.4				0.716	0.811	-0.084
Pain							0.798			
Pre	18.4 ± 26.1			21.6 ± 25.9				0.592	0.910	-0.124
1 week	31.7 ± 21.8			30.4 ± 24.7				0.813	0.910	0.055
2 weeks	18.2 ± 18.2			18.7 ± 21.3				0.910	0.910	-0.026
3 weeks	12.9 ± 13.9			10.9 ± 14.2				0.524	0.910	0.148

Mean ± standard deviation (SD). ROM: range of motion, TUG: timed up and go test

*false discovery rate adjusted p-value for multiple testing using the Benjamini-Hochberg method.

## Discussion

The purpose of this study was to assess the beneficial effects of SST training, which focuses on the quickness of trunk movement, on mobility in patients who had undergone TKA. As predicted, SST training produced a significant improvement in mobility as represented by gait speed and TUG time over the first 3 weeks after TKA, but no significant difference was observed in postoperative improvement in knee function, represented by knee range of motion and quadriceps and hamstrings muscle strengths. The differences in gait speed between the groups with and without SST training from the first to the third week after TKA were 0.13, 0.11, and 0.11 m/s, respectively. These values all exceed 0.1 m/s, which is generally accepted as a clinically relevant difference [21, 22]. We previously demonstrated that SST is a good indicator for predicting mobility in older people. Furthermore, this study suggests that SST training is an effective intervention with which to improve mobility in patients who have undergone TKA.

One of the main purposes of performing TKA is to improve mobility [4]. However, the mobility of patients who have undergone TKA is often considerably attenuated at 1 month after surgery as compared with the baseline level [5]. Moreover, even at 1 year after TKA, gait speed is not always fully recovered in older adults [6].

Previous studies reported that the gait speed after TKA improved nonlinearly over time with a steep improvement rate during the first 8 to 12 weeks and beyond, as the improvement over time was more gradual [25, 26, 27]. Moreover, other studies showed that new-concept rehabilitation programs (early high-intensity rehabilitation or early neuromuscular electrical stimulation) could improve gait function for the first 8 weeks after TKA, with its benefits persisting for 1 year [28, 29]. In these studies, the substantial improvement of gait function was observed within the first 10 weeks and the degree of improvement in this period had long-term effects. Compared with these previous studies, the intervention and observation periods in the present study were short. Therefore, how long the improvement of gait function obtained with SST training would last is not clear. However, this study shows, at least, that our intervention term was within an important period for the improvement of gait function and that SST training is an effective intervention during this period.

The significant improvement in the mobility of the patients who received TKA may be attributable mainly to two factors. First, trunk function is essential for mobility in patients who receive TKA because trunk muscle action can compensate for knee function during walking [12]. Trunk muscle endurance and strength are associated with both balance and mobility performance in mobility-limited older adults [30]. Furthermore, trunk control in a lateral direction is necessary for maintaining balance during walking [10]. The ability to move the trunk in any direction indicates mobility in healthy older people [31]. In both observational and interventional studies, trunk muscle exercises were shown to be useful for improving mobility. Trunk exercises, which include strength and coordination, can be beneficial for improving the mobility function of people who had a stroke [32]. Both isometric and dynamic resistance trunk exercises have been shown to increase gait velocity in community-dwelling older people [33]. As SST training consists of quick lateral movements in a seated position, the training is aimed at improving trunk function. Therefore, it is conceivable that improvement of trunk function achieved with SST training led to the improvement of mobility after TKA.

Second, movement velocity is crucial to determine the mobility of older people. The association between the maximum angular velocity of knee extension and gait speed is significant in community-dwelling older people [34]. The movement velocity of the upper limbs was also significantly associated with gait speed and TUG time in our previous study [35]. In addition to these cross-sectional studies, a number of intervention studies have focused on movement velocity. As compared with low-speed resistance training, high-speed resistance training was reported to improve gait speed and TUG time in healthy older women [14]. Recently, high-velocity training, even though without load, has been reported to improve the mobility of community-dwelling older adults [36]. These previous studies showed that training focused on movement velocity is highly effective for improving the mobility of older subjects. As SST training was performed without external loads and with maximum speed, we assumed that the movement velocity of the trunk was improved with the training and led to the improvement of mobility after TKA in the present patients.

SST training has several advantages to the conventional training methods because it is safe and easy to perform. As the patients are seated, they are not at risk of falling and have no fear of falling or pain. In fact, none of the participants reported any pain during training in this study. The time needed for this training was short. The average time of one set of training was <10 seconds, and the total duration of one training session was <3 minutes, including preparation and rest between the sets. In addition, the intensity of SST training was low. The participants could generally perform SST training in addition to normal exercise without fatigue. Furthermore, it does not require special techniques, experience, or apparatus, so it can be performed without any prior experience.

Some limitations of the study should be noted. The first and major limitation is that this study could not demonstrate the long-term effects (>3 weeks) of SST training on mobility after TKA. Second, the possibility of a type II statistical error cannot be entirely excluded. Although an a priori power analysis suggested that the sample size was adequate, a post hoc power analysis revealed that it was smaller than necessary to achieve 80% power for gait speed at 2 and 3 weeks and TUG time at 1 week. Third, because the measurements in the mobility tests were performed by non-blinded evaluators, we considered the possibility of evaluator bias influencing the results of this study. Finally, we did not evaluate which components of SST training contributed to the beneficial effects. To move the trunk quickly, not only the trunk but also the lower and upper limbs are needed. Further research is required to determine which component(s) of SST training actually produces an improvement in mobility.

In conclusion, SST training produced significant and clinically relevant improvements in mobility during 3 weeks after TKA. This result indicates that quick lateral trunk movement training is a useful intervention after TKA to improve mobility in the short term, despite no difference in knee function, including knee pain. As SST training is easy and safe, and requires no special techniques or apparatus, it can be recommended for inclusion in rehabilitation programs where the aim is to improve mobility after TKA. However, because we could not determine the long-term effects of SST training, further research is required in this area.

## Supporting information

S1 Table Trial study protocol. (DOC)

S1 File Trial study protocol (Japanese edition). (DOC)

S1 Fig CONSORT checklist. (DOC)
