# Supplementary material for: An easy and safe training method for trunk function improves mobility in total knee arthroplasty patients: A quasi-randomized controlled trial
Source: PLoS One. 2018 Oct 4;13(10):e0204884. doi: 10.1371/journal.pone.0204884 (PMC6171877; doi:10.1371/journal.pone.0204884)
Supplement: S1 Table — (DOC) [file pone.0204884.s001.doc]

Trial study protocol

| Title | An Easy and Safe Training Method for Trunk Function Improves Mobility in Total Knee Arthroplasty Patients |
| --- | --- |
| Summary | The aim of this trial is to investigate the effectiveness of Seated Side Tapping (SST) training (3 minutes including preparations per day, 5 days per week, for 3weeks), which focuses on the quickness of trunk movement in a seated position, on mobility (gait speed and TUG) in TKA patients. |
| Study Design | Quasi-randomized controlled trial  Non-blinded study |
| Description | We previously showed that the seated side tapping (SST) test, which measures the time in seconds needed to move the body laterally from one side to the other ten times as quickly as possible in a seated position, was closely associated with mobility in frail and healthy elderly subjects.  **Purpose**  The purpose of this study is to assess the effects of SST as a training program (“SST training”) on the prognosis of mobility in Total Knee arthroplasty (TKA) patients.  **Participants**  One hundred participants, who are scheduled to undergo TKA due to knee OA at Osaka General Medical Center, will be recruited. Participants will be randomly assigned to the SST group or control group using a quasi-randomized design in order of operation.  **Overview of intervention**  A standard rehabilitation program performed 5 days a week by a physical therapist will be initiated the day after TKA and continue for three weeks. Participants in the SST group will carry out SST training in addition to the standard inpatient rehabilitation program 5 days per week, for 3weeks. The apparatus of SST training is that the therapists place a stand on either side of the chair that the patient is sitting on, and a marker was placed on each stand. The participants raise their arms to shoulder height, and the therapist move the stands to positions 10 cm away from the tips of their fingers. The participants are instructed to tap the markers in turn 10 times as quickly as possible. One set of SST training includes 5 repetitions with approximately 10 seconds intervals and the total time of one set of training including preparation is 3 minutes per day.  The procedures for this study have been approved by the ethics committees of Osaka Prefecture University and Osaka General Medical Center. |
| Inclusion/Exclusion criteria | Inclusion criteria:   1. Patients who have total knee arthroplasty and total hip arthroplasty 2. 60 years-old or older 3. Patients who have the ability to walk 10 m or more without assistance one week after surgery.   Exclusion criteria:  They have any medical or neurological problem that would affect their ability to complete this trial, such as severe dementia, stroke, cardiac insufficiency, or acute respiratory failure. |
| Primary Outcome Measures | To evaluate the effect of the SST training on mobility (gait speed and TUG) after TKA patient. |
| Secondary Outcome Measures | Range of Motion (knee flexion and extension), Pain (VAS), Muscle Strength (knee flexion and extension) |
| Sample size | 100 |
| Setting | Osaka city, Osaka prefecture in Japan |
| Project Coordinator | Yuki Sano  Researcher,  Department of Rehabilitation, Osaka General Medical Center, Bandaihigashi 3-1-56, Osaka Sumiyoshi-ku, Osaka, Japan |
| Overall Study Official | Department of Physical Therapy, Faculty of Comprehensive Rehabilitation, Osaka Prefecture University, Habikino 3-7-30, Osaka, Japan |
| Human Subjects Review/  Oversight | This research was approved by the Ethics Committees of Osaka Prefecture University and Osaka General Medical Center. |
| Collaborators | Akira Iwata^1^, Hideyuki Wanaka^1,2^, Mina Matsui^2^, Saki Yamamoto^1^, Junichiro Koyanagi^3^, Hiroshi Iwata^4^  1 Department of Physical Therapy, Faculty of Comprehensive Rehabilitation, Osaka Prefecture University, Habikino 3-7-30, Osaka, Japan  2 Department of Rehabilitation, Osaka General Medical Center, Bandaihigashi 3-1-56, Osaka Sumiyoshi-ku, Osaka, Japan  3 Department of Orthopaedic Surgery, Osaka General Medical Center, Bandaihigashi 3-1-56, Osaka Sumiyoshi-ku, Osaka, Japan  4 Department of Cardiovascular Medicine, Juntendo University Graduate School of Medicine, Bunkyo-ku, Tokyo, Japan |
| Funding Source | This work is supported by JSPS KAKENHI Grant Number 26350628. https://www. jsps.go.jp/english/index.html. |
| References | 1. Bade MJ, Kohrt WM, Stevens-Lapsley JE. Outcomes before and after total knee arthroplasty compared to healthy adults. J Orthop Sports Phys Ther. 2010; 40(9): 559-67. doi: [10.2519/jospt.2010.3317](https://dx.doi.org/10.2519/jospt.2010.3317) PMID: 20710093 2. Walsh M, Woodhouse LJ, Thomas SG, Finch E. Physical impairments and functional limitations: a comparison of individuals 1 year after total knee arthroplasty with control subjects. Phys Ther. 1998; 78(3): 248-58. doi: [10.1093/ptj/78.3.248](https://doi.org/10.1093/ptj/78.3.248) PMID: 9520970 3. Helbostad JL, Moe-Nilssen R. The effect of gait speed on lateral balance control during walking in healthy elderly. Gait Posture. 2003; 18(2): 27-36. doi: [10.1016/S0966-6362(02)00197-2](http://dx.doi.org/10.1016/S0966-6362(02)00197-2) PMID: 14654205 4. Sayers SP, Guralnik JM, Thombs LA, Fielding RA. Effect of leg muscle contraction velocity on functional performance in older men and women. J Am Geriatr Soc. 2005; 53(3): 467-71. doi: [10.1111/j.1532-5415.2005.53166.x](https://dx.doi.org/10.1111/j.1532-5415.2005.53166.x) PMID: 15743291 5. Higuchi Y, Iwata A, Fuchioka S. [Lateral trunk control in a sitting test is associated with mobility and Instrumental Activities of Daily Living among community-dwelling elderly people, in press]. Nihon Ronen Igakkai Zasshi. 2012; 49(4): 449-56. doi: [10.3143/geriatrics.49.449](http://doi.org/10.3143/geriatrics.49.449) PMID: 23269024 |
