# Supplementary material for: An easy and safe training method for trunk function improves mobility in total knee arthroplasty patients: A quasi-randomized controlled trial
Source: PLoS One. 2018 Oct 4;13(10):e0204884. doi: 10.1371/journal.pone.0204884 (PMC6171877; doi:10.1371/journal.pone.0204884)
Supplement: S1 File — (DOC) [file pone.0204884.s002.doc]

自主臨床研究

坐位での側方移動トレーニングが
歩行能力に及ぼす影響について

**研　究　実　施　計　画　書**

研究責任者 大阪府立急性期・総合医療センター

リハビリテーション科　　佐野　佑樹

**1．研究の背景**

　　本研究は変形性関節症（OA：osteoarthrosis）を対象とする。一般にOAは、加齢、性別（女性）、肥満、過度の負荷などがリスクファクターとして挙げられる。発症頻度と年齢は、男女比で1～1.5：10、女性は男性に比べ8～10年早い40代後半から50代前半で発症する^1)^。また、大規模臨床統合データベースROAD（Research on Osteoarthritis Against Disability）の調査から、我が国の中高年における膝OAの有病率は男性42.0％、女性61.5％で有り、40歳以上の膝OA患者数を推定すると、2350万人（男性860万人、女性1670万人）と累計されている。膝OAに対しては、保存的治療と手術治療が考慮される。中でも、人工関節全置換術は、我が国では年間10万件程度行われていると言われている^2)^。下肢の人工関節の手術の代表的なものが、人工膝関節全置換術（TKA：total knee arthroplasty）である。TKA術前後のリハビリテーション（以下リハ）は、クリニカルパスに沿って行われており、通常術後3～4週間程度で退院となる^3)^。その中で、歩行能力の向上は、TKA術後のリハにおいて非常に重要な要素である^4)^。術前後の歩行能力の評価項目としては，Timed Up & Go test（TUG）やFunctional reach（FR），タンデム肢位での重心動揺，最大歩行速度などが報告されている^5),6),7)^が，いずれも立位や歩行を行う必要があり，術前の患者では疼痛が強く，正確な歩行能力を評価しているとは言えないのではないかと考えられる。そこで、坐位で簡便に歩行能力が評価でき、健常高齢者や虚弱高齢者の歩行能力や立位パフォーマンスとの関連が深いSeated Side Tapping test（SST）^8),9)^を、整形外科疾患にも適応できないかと考えた。SSTを通常の治療プログラムに取り込む対象群と、通常の治療のみ行う比較対照群とを比較することで、SSTの治療効果についても言及が可能になる。

**2．研究の目的**

SSTが整形外科疾患患者の術後の歩行能力の予後予測として使える指標であるか、またSSTを術後の治療プログラムへ導入すれば，早期の回復が期待できるため，その介入効果についても検討する。

**3．対象患者および適格性の基準**

（1）対象患者のうち、（2）選択基準をすべて満たし、かつ（3）除外基準のいずれにも該当しない場合を適格とする。

（1）対象患者

大阪府立急性期・総合医療センター整形外科に入院し、TKA術前後の患者を対象とする。

（2）選択基準

①同意取得時において年齢が60歳以上90歳未満の患者

②術前にADLが比較的自立できていた症例を対象とする。

③本研究への参加にあたり十分な説明を受けた後、十分な理解の上、患者本人の自由意思による文書同意が得られた患者

（3）除外基準

①クリニカルパスから逸脱した症例は除外する

②中等度異常の呼吸・循環器疾患を有する場合、維持透析中、認知症などを有し理解力の低下を認める症例、脊髄症や脳血管障害により運動・感覚麻痺を呈している症例は除外する

③その他、研究責任者が被験者として不適当と判断した患者

**4．研究の方法**

（1）研究の種類・デザイン

対照、ランダム化、非盲検、並行群間比較

（2）研究のアウトライン

　　　リハビリ依頼が来た時点で、SSTの対象群と比較対照群とに割付を行う。手術前日、同意を取得し、術前評価を実施する。手術後2日目より対象群はSST開始し、退院前日まで行う。また対象群、比較対照群ともに手術後7日目、14日目、退院前日にSSTと合わせて以下の検査を行う。

（3）観察・及び検査項目

①患者基本情報

年齢、性別、身長、体重、診断名

②SST

　椅子に坐った状態で、左右に置かれたボタンを交互に10回押し、それに要した時間を計測

③10m歩行速度

快適歩行速度を計測

④TUG：Timed Up & Go test

　椅子に坐った状態から3m先のマークを回って帰ってくるまでに要した時間
を計測

⑤VAS：Visual analog scale

⑥筋力：膝関節伸展

⑦関節可動域

＊観察および検査スケジュール表

SST対象群

|  | 観察期間 （3週間） | | | | |
| --- | --- | --- | --- | --- | --- |
| 期間 | 術前 | 術後2d | 7d | 14d | 退院前日 |
| 同意 | ○ |  |  |  |  |
| 患者基本情報 | ○ |  |  |  |  |
| SST | ○ | ←　○　→ | | | |
| 10m歩行速度 | ○ |  | ○ | ○ | ○ |
| TUG | ○ |  | ○ | ○ | ○ |
| VAS | ○ |  | ○ | ○ | ○ |
| 筋力 | ○ |  | ○ | ○ | ○ |
| 関節可動域 | ○ |  | ○ | ○ | ○ |
| 有害事象 |  | ←　○　→ | | | |

　　比較対照群

|  | 観察期間 （3週間） | | | | |
| --- | --- | --- | --- | --- | --- |
| 期間 | 術前 | 術後2d | 7d | 14d | 退院前日 |
| 同意 | ○ |  |  |  |  |
| 患者基本情報 | ○ |  |  |  |  |
| SST | ○ |  | ○ | ○ | ○ |
| 10m歩行速度 | ○ |  | ○ | ○ | ○ |
| TUG | ○ |  | ○ | ○ | ○ |
| VAS | ○ |  | ○ | ○ | ○ |
| 筋力 | ○ |  | ○ | ○ | ○ |
| 関節可動域 | ○ |  | ○ | ○ | ○ |
| 有害事象 |  | ←　○　→ | | | |

（4）症例登録、割付の方法

新規のリハビリ依頼が来た時点で、同意を取得し症例を登録し、登録順に対象群、比較対照群に割付する。

（5）被験者の研究参加予定期間

同意取得後から退院までの約3週間程度とする。

（6）研究終了後の対応

本研究終了後は、この研究で得られた成果も含めて、研究責任者は被験者に対し最も適切と考える医療を提供する。

**5．予想される利益および不利益（副作用）**

（1）予想される利益

・SSTの訓練効果による、歩行速度の改善が得られる可能性がある。

・研究成果により将来の医療の進歩に貢献できる可能性がある。

（2）予想される不利益（副作用）

・研究実施に伴う10分程度の時間的拘束が発生するが、特にこの研究に参加することにより、新たな不利益は生じない

**6．評価項目（エンドポイント）**

（1）主要評価項目

SST対象群と比較対照群との歩行速度の変化

（2）副次的評価項目

ROM，筋力，疼痛と歩行機能の関係

**7．個々の被験者における中止基準**

（1）研究中止時の対応

研究責任者または研究分担者（以下、研究担当者）は、次に挙げる理由で個々の被験者について研究継続が不可能と判断した場合には、当該被験者についての研究を中止する。その際は、必要に応じて中止の理由を被験者に説明する。また、中止後の被験者の治療については、被験者の不利益とならないよう、誠意を持って対応する。

（2）中止基準

①被験者から研究参加の辞退の申し出や同意の撤回があった場合

②クリニカルパスから逸脱した場合

③転倒等に伴うインシデントが発生した場合

④本研究全体が中止された場合

⑤その他の理由により、研究担当者が研究の中止が適当と判断した場合

**8．有害事象発生時の取扱い**

（1）有害事象発生時の被験者への対応

研究担当者は、有害事象を認めたときは、直ちに適切な処置を行うとともに、診療録ならびに症例報告書に記載する。また、介入行為を中止した場合や、有害事象に対する治療が必要となった場合には、被験者にその旨を伝える。

（2）重篤な有害事象の報告

　重篤な有害事象は、薬事法施行規則第273条に準じて次の通りに定義する。

①死亡または死亡につながるおそれ

②入院または入院期間の延長

③障害または障害につながるおそれ

④後世代または先天性の疾病または異常

　研究責任者は、研究期間中の全ての重篤な有害事象、研究終了（中止）後に介入行為との関連性が疑われる重篤な有害事象について、速やかに自主臨床研究事務局を通じて病院長に報告する。報告は、自主臨床研究標準業務手順書に準じて、第一報（緊急報告）および第二報以降（詳細報告）とする。

（3）重要な有害事象の報告

　　　　該当なし

（4）その他の有害事象

　その他の有害事象については、研究担当者は適切に診療録および症例報告書に記載する。

**9．研究実施計画書等の変更**

本研究の研究実施計画書や同意説明文書の変更または改訂を行う場合は、あらかじめ臨床医学倫理委員会（以下、審査委員会）の承認を必要とする。

**10．研究の変更、中止・中断、終了**

（1）研究の変更

本研究の研究実施計画書や同意説明文書の変更または改訂を行う場合は、あらかじめ審査委員会の承認を必要とする。

（2）研究の中止、中断

研究担当者は、以下の事項に該当する場合は、研究実施継続の可否を検討する。

①被験者の組み入れが困難で、予定症例数に達することが極めて困難であると判断されたとき。

②予定症例数または予定期間に達する前に、研究の目的が達成されたとき。

③審査委員会により、実施計画等の変更の指示があり、これを受入れる
ことが困難と判断されたとき。

研究責任者は、審査委員会により中止の勧告あるいは指示があった場合は、研究を中止する。また、研究の中止または中断を決定した時は、速やかに院長にその理由とともに文書で報告する。

（3）研究の終了

　研究の終了時には、研究責任者は速やかに研究終了報告書を院長に提出する。

**11．研究実施期間**

2012年1月1日～2014年3月31日

**12．目標症例数とその設定根拠および統計解析方法**

（1）目標症例数とその設定根拠

100例

【設定根拠】

　　　　　当院整形外科ではTKAは年間200例以上の手術が行われている。また淵岡らによるSSTを用いた先行研究^8),9)^では、およそ100例程度の症例数で行われており、十分実施可能な症例数と考えられる。

（2）統計解析方法

対象群と、比較対照群の2群間比較をt検定で行う。今後研究の進捗により、適切な検定を選択する可能性もある。

**13．被験者の人権に対する配慮および個人情報の保護の方法**

本研究のすべての担当者は、「ヘルシンキ宣言（2008年10月修正）」および「臨床研究に関する倫理指針（平成20年7月31日改正、以下臨床研究倫理指針）」を遵守して実施する。

研究実施に係る試料等を取扱う際は、被験者の個人情報とは無関係の番号を付して管理し、被験者の秘密保護に十分配慮する。試料等を研究事務局等の関連機関に送付する場合はこの番号を使用し、被験者の個人情報が院外に漏れないよう十分配慮する。また、研究の結果を公表する際は、被験者を特定できる情報を含まないようにする。研究の目的以外に、研究で得られた被験者の試料等を使用しない。

**14．同意取得方法**

研究担当者は、審査委員会で承認の得られた同意説明文書を被験者に渡し、文書および口頭による十分な説明を行い、被験者の自由意思による同意を文書で取得する。

研究担当者は、被験者の同意に影響を及ぼす情報が得られたときや、被験者の同意に影響を及ぼすような実施計画等の変更が行われるときは、速やかに被験者に情報提供し、研究に参加するか否かについて被験者の意思を予め確認するとともに、事前に審査委員会の承認を得て同意説明文書等の改訂を行い、被験者の再同意を得ることとする。

同意説明文書には、以下の内容を含むものとする。

①研究への参加は任意であること、同意しなくても不利益を受けないこと、同意は撤回できること

②研究の意義（背景）、目的、対象、方法、実施期間、予定被験者数

③研究に参加することにより期待される利益、起こりえる不利益

④個人情報を含めた試料等の取扱い、保存期間と廃棄方法、研究方法等の閲覧

⑤研究成果の発表および特許が発生した場合の取扱い

⑥研究に係る被験者の費用負担、研究資金源と利益相反

⑦研究の組織体制、研究に関する問い合わせ、苦情等の相談窓口（連絡先）

⑧被験者に健康被害が発生した場合の対応と補償の有無

**15．被験者の健康被害への対応と補償**

本研究の実施に伴い、被験者に健康被害が発生した場合は、研究担当者は適切な処置を講じる。その際、治療または検査等が必要となった場合は、被験者の通常の保険診療内で実施する。この点を被験者に説明し、理解を得ることとする。

**16．被験者の費用負担**

本研究で実施する行為は保険診療内で行われるため、研究に参加することによる患者の費用負担は発生しない。

**17．記録の保存と研究結果の公表**

研究責任者は、研究等の実施に係わる重要な文書（申請書類の控え、病院長からの通知文書、各種申請書・報告書の控、同意書、その他データの信頼性を保証するのに必要な書類または記録等）を、研究の中止または終了後3年が経過した日までの間保存し、その後は個人情報に注意して廃棄する。

研究担当者は、本研究の成果を関連学会等において発表することにより公表する。

**18．研究資金および利益相反**

本研究は、研究責任者が所属する診療科の研究費で実施する。また、本研究の研究担当者は、利益相反マネジメント審査委員会に必要事項を申告し、その審査と承認を得るものとする。

**19．研究実施体制**

本研究は以下の体制で実施する。

【研究責任者】

佐野　佑樹 大阪府立急性期・総合医療センター　リハビリテーション科

岩田　晃 大阪府立大学大学院総合リハビリテーション学研究科

【研究分担者】

和中　秀行 大阪府立急性期・総合医療センター　リハビリテーション科

松井　未衣菜 大阪府立急性期・総合医療センター　リハビリテーション科

山本　沙紀 大阪府立大学大学院総合リハビリテーション学研究科

小柳　淳一郎 大阪府立急性期・総合医療センター　整形外科

岩田　洋 順天堂大学大学院医学研究科　循環器内科

**20．参考資料・文献リスト**

1. Bade MJ, Kohrt WM, Stevens-Lapsley JE. Outcomes before and after total knee arthroplasty compared to healthy adults. J Orthop Sports Phys Ther. 2010; 40(9): 559-67. doi: [10.2519/jospt.2010.3317](https://dx.doi.org/10.2519/jospt.2010.3317) PMID: 20710093
2. Walsh M, Woodhouse LJ, Thomas SG, Finch E. Physical impairments and functional limitations: a comparison of individuals 1 year after total knee arthroplasty with control subjects. Phys Ther. 1998; 78(3): 248-58. doi: [10.1093/ptj/78.3.248](https://doi.org/10.1093/ptj/78.3.248) PMID: 9520970
3. Helbostad JL, Moe-Nilssen R. The effect of gait speed on lateral balance control during walking in healthy elderly. Gait Posture. 2003; 18(2): 27-36. doi: [10.1016/S0966-6362(02)00197-2](http://dx.doi.org/10.1016/S0966-6362(02)00197-2) PMID: 14654205
4. Sayers SP, Guralnik JM, Thombs LA, Fielding RA. Effect of leg muscle contraction velocity on functional performance in older men and women. J Am Geriatr Soc. 2005; 53(3): 467-71. doi: [10.1111/j.1532-5415.2005.53166.x](https://dx.doi.org/10.1111/j.1532-5415.2005.53166.x) PMID: 15743291
5. Higuchi Y, Iwata A, Fuchioka S. [Lateral trunk control in a sitting test is associated with mobility and Instrumental Activities of Daily Living among community-dwelling elderly people, in press]. Nihon Ronen Igakkai Zasshi. 2012; 49(4): 449-56. doi: [10.3143/geriatrics.49.449](http://doi.org/10.3143/geriatrics.49.449) PMID: 23269024
